# Endoplasmic reticulum stress and inflammation in the central nervous system

**DOI:** 10.1186/s13024-017-0183-y

**Published:** 2017-05-25

**Authors:** Neil T. Sprenkle, Savannah G. Sims, Cristina L. Sánchez, Gordon P. Meares

**Affiliations:** 1 Department of Microbiology, Immunology and Cell Biology, West Virginia University School of Medicine, One Medical Center Drive, BMRC, Morgantown, WV 311 USA; 2 Blanchette Rockefeller Neurosciences Institute, West Virginia University School of Medicine, Morgantown, WV USA

**Keywords:** Endoplasmic reticulum stress, Unfolded protein response, Neuroinflammation, Neurodegeneration

## Abstract

Persistent endoplasmic reticulum (ER) stress is thought to drive the pathology of many chronic disorders due to its potential to elicit aberrant inflammatory signaling and facilitate cell death. In neurodegenerative diseases, the accumulation of misfolded proteins and concomitant induction of ER stress in neurons contributes to neuronal dysfunction. In addition, ER stress responses induced in the surrounding neuroglia may promote disease progression by coordinating damaging inflammatory responses, which help fuel a neurotoxic milieu. Nevertheless, there still remains a gap in knowledge regarding the cell-specific mechanisms by which ER stress mediates neuroinflammation. In this review, we will discuss recently uncovered inflammatory pathways linked to the ER stress response. Moreover, we will summarize the present literature delineating how ER stress is generated in Alzheimer’s disease, Parkinson’s disease, Amyotrophic Lateral Sclerosis, and Multiple Sclerosis, and highlight how ER stress and neuroinflammation intersect mechanistically within the central nervous system. The mechanisms by which stress-induced inflammation contributes to the pathogenesis and progression of neurodegenerative diseases remain poorly understood. Further examination of this interplay could present unappreciated insights into the development of neurodegenerative diseases, and reveal new therapeutic targets.

## Background

Innate immune activation has emerged as a prominent component in the pathology of many neurodegenerative diseases. Previously, the involvement of immunity in the pathogenesis of neurological disorders had been greatly underappreciated. However, within the last couple decades we have come to realize that an aberrant inflammatory program within the central nervous system (CNS) contributes to neuronal dysfunction [1]. While inflammation is considered a beneficial physiological response, as it promotes debris clearance and aids in tissue repair, sustained inflammatory signaling overwhelms the resolution capabilities of the CNS [2]. This, in turn, is thought to be fundamental to the development of harmful neuroinflammation. Brain-resident microglia and astrocytes are the main source of inflammation in the brain, and under pathological conditions these dysregulated glial cells facilitate the events that promote a neurotoxic microenvironment [3, 4]. Considering that neurons have a limited regenerative capacity, excessive neuronal loss in the CNS has dire consequences on motor and cognitive function. A wealth of data now supports the hypothesis that inflammation in the CNS may contribute to neurodegeneration by establishing a feed-forward inflammatory loop which ultimately leads to sustained neuronal damage [1, 2, 5, 6]. Importantly, this likely reflects impairment of the normal mechanisms involved in immune responses in the brain as inflammation, glial activation and even peripheral immune infiltration are essential elements of normal tissue homeostasis and repair [7, 8].

One of the pathological hallmarks of many neurodegenerative diseases is the accumulation of misfolded proteins within the ER of neurons and neuroglia. In response to ER stress, cells induce a highly conserved cellular stress response called the unfolded protein response (UPR) in an attempt to maintain homeostasis [9]. The UPR program orchestrates transcriptional and translational changes in the cell to minimize stress, while concomitantly inducing protein quality control mechanisms in an attempt to reduce protein misfolding. If resolution fails, the temporally-regulated induction of UPR-dependent inflammatory and apoptotic pathways has the potential to exacerbate neuroinflammation and compromise cell fidelity [10–13].

Accumulating evidence suggests that cells under severe ER stress caused by various insults interfere with the immunosuppressive environment of the CNS [10, 11, 14]. These findings imply a heterogeneous cause linking ER stress in neurons, microglia and astrocytes with inflammation in the progression of neurodegeneration. Novel intracellular processes involved in this integrative cellular response continue to emerge. Here, we will introduce recently discovered signaling pathways associated with the UPR and present current findings regarding how chronic ER stress engenders neurological abnormalities. Furthermore, we will discuss how a UPR-induced inflammatory phenotype in CNS-resident cells could promote conditions responsible for impairing neuronal function.

## The physiological role of the UPR

The majority of proteins destined for the secretory pathway present a hydrophobic N-terminal signal sequence during the initial stages of translation [15, 16]. This amino acid sequence is recognized by the cytosolic protein signal-recognition particle, which coordinates cotranslational translocation of the nascent polypeptide across the ER membrane and into the ER lumen [17, 18]. Here, the unique environment of the ER lumen facilitates the proper folding events that create a stable protein with functional capabilities.

The ER serves as the cell’s largest calcium store owing to the consistent active transport of calcium into the lumen [19]. Intraluminal ER calcium is necessary for the activation of calcium-dependent molecular chaperones, including the ER resident glucose-regulated proteins (GRPs), which go on to stabilize protein folding intermediates [20]. Furthermore, the ER lumen possesses an oxidative environment which allow protein disulphide isomerases (PDIs) to catalyze the formation of disulfide bonds. Reduced PDIs are reoxidized by endoplasmic reticulum oxidoreductase α (Ero1α) to allow for continuous oxidation of free cysteine residues residing on unfolded proteins [21]. Additional post-translational modifications, such as glycosylation, are executed within the ER to produce a mature protein that is packaged into coat protein complex II-coated vesicles and exported out of the ER [22, 23]. ER-derived vesicles then enter the canonical secretory pathway where cargo is either targeted to the plasma membrane or to other cellular compartments.

Features of pathophysiological stress in the form of gene mutations, protein aggregates, inflammatory signals, metabolic alterations, pathogen-associated molecular patterns (PAMPs), danger-associated molecular pattern molecules (DAMPs) and/or reactive oxygen or nitrogen species (ROS/RNS) disrupts efficient protein folding processes in the ER lumen, thus creating an imbalance between the protein load and the folding capabilities of the ER [24]. The UPR responds to cellular stress by triggering effector mechanisms that can be grouped as adaptive, alarming or pro-apoptotic [20]. In the adaptive phase of the UPR, mammalian cells are able to tolerate moderate protein misfolding by upregulating the expression of chaperone proteins to correctly fold and stabilize the increasing amount of polypeptide transported into the ER lumen. In an effort to maintain quality control, the cell also employs ER-associated degradation (ERAD) and attenuates translation of global messenger RNA (mRNA) to alleviate the protein load within the lumen [25, 26]. In more severe situations, the protein folding capacity of the ER fails to keep pace with the increasing influx of polypeptide, as the extensive accumulation of misfolded proteins in the ER lumen begins to overwhelm the compensatory mechanisms of the UPR. If improperly regulated, the buildup of misfolded proteins will compromise normal cellular processes. Under these conditions, the cell initiates signaling pathways associated with cellular stress, most notably the activation of inflammatory pathways, and ‘alarms’ the extracellular environment of the distress so that the appropriate tissue-wide response is initiated [20]. If all else fails the UPR will trigger cell death through both caspase-dependent and -independent means [27–29].

### Signal transducers of the ER stress response

**Fig. 1:**
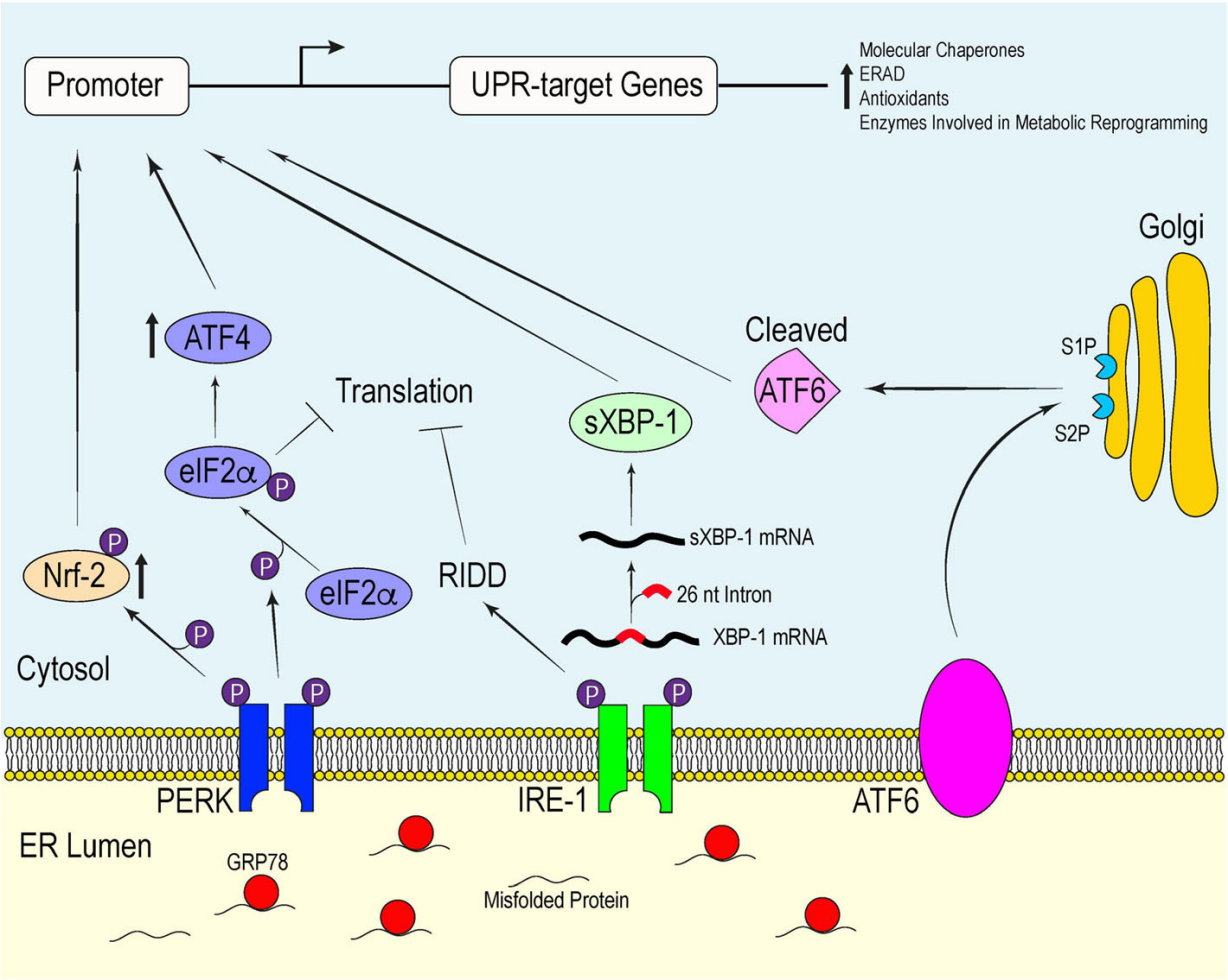
The Adaptive Signals of the Mammalian UPR. The activation of PERK, IRE-1α and ATF6 in response to protein misfolding stress primarily requires the dissociation of the molecular chaperone GRP78 from each of the ER stress sensors. This initiates signaling cascades which orchestrate the transcriptional and translational landscape of the cell in an effort to maintain homeostasis

In mammalian cells, the central proteins involved in initiating this evolutionarily conserved response are activating transcription factor 6 (ATF6), inositol-requiring 1α (IRE-1α) and double-stranded RNA dependent protein kinase-like ER kinase (PERK) [24]. GRP78 (also known as binding immunoglobulin protein (BiP)) primarily regulates the initiation of the UPR through its direct interactions with each signal transducing sensor [30–32] (Fig. 1). Physical contact between GRP78 and the luminal domain of the ER-transmembrane proteins stabilizes their inactive state. High demand for chaperone-mediated protein stabilization brought on by increases in protein synthesis or defective protein folding recruits GRP78 away from these proteins [31]. Disrupting this interaction frees the luminal domain of the ER sensors, consequently inducing their functional conformation. Recent evidence has suggested an additional regulatory mechanism by which the sensors become catalytically active. By crystallizing the yeast IRE-1, Credle et al. elucidated a distinct peptide-binding groove in the IRE-1 luminal domain [33, 34]. In this model, unfolded polypeptide within the ER lumen may act as a substrate for the peptide-binding groove located in IRE-1. Because of the shared structural homology with that of IRE-1, PERK may also be activated in a similar manner [33]. These findings represent a unique sensing mechanism that regulates the activation of the UPR.

**Fig. 2:**
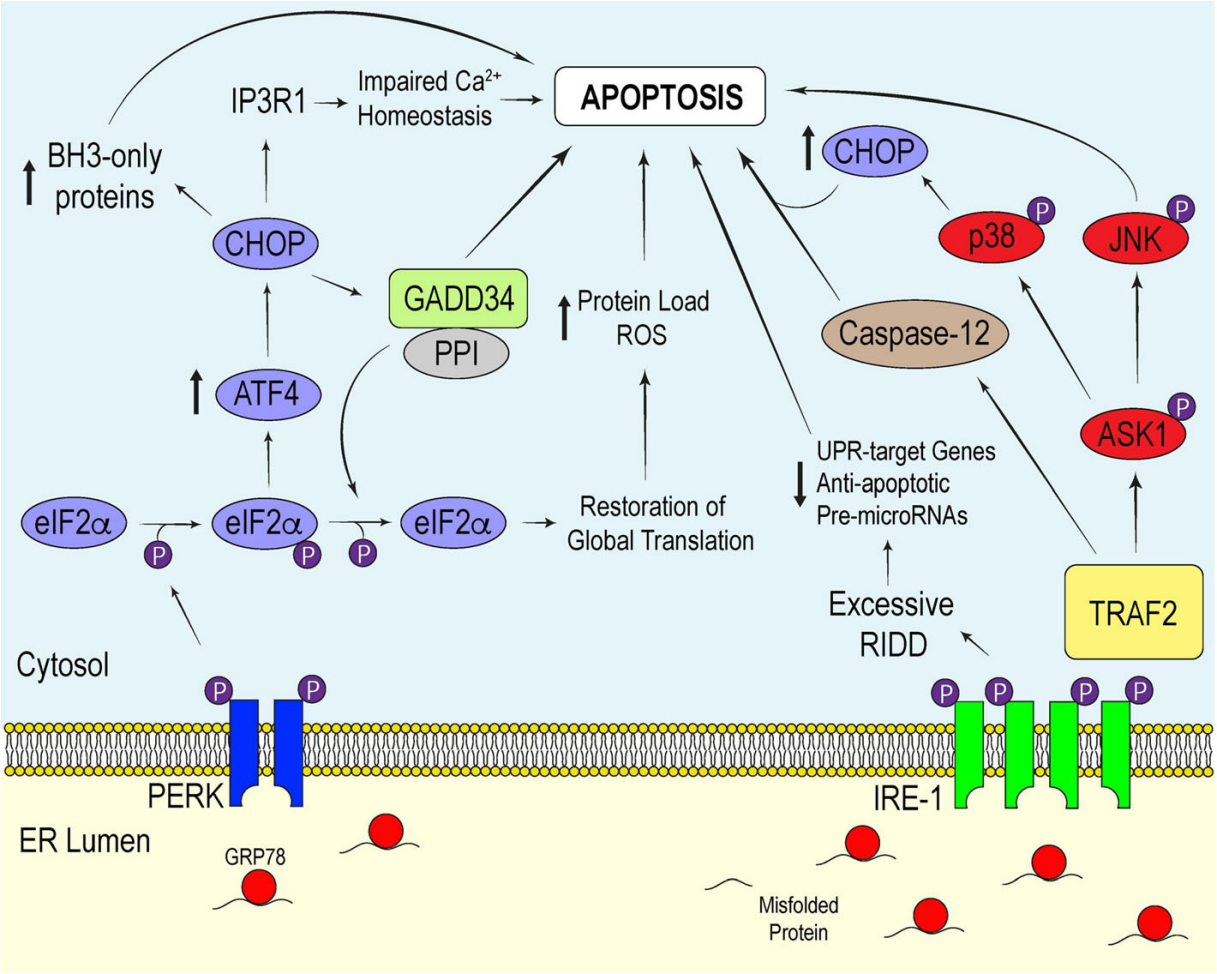
Apoptotic Signals Associated with Chronic UPR Activation. Persistent ER stress triggers the apoptotic component of the UPR. PERK and IRE-1α drive UPR-induced apoptosis by initiating pathways which facilitate enhanced ROS production, Ca^2+^ dysregulation and caspase activation

Interplay between active ATF6, IRE-1α and PERK initiate signaling cascades that regulate the transcriptional and translational landscape of the cell to selectively promote the expression UPR-target genes. Each of these mediators promote distinct signaling pathways which converge to produce an effective response to mitigate damage. If overwhelmed, these signaling proteins will initiate apoptosis [35] (Fig. 2).

Mammalian ATF6 is a type II transmembrane protein embedded within the ER membrane [36]. The carboxyl terminus of ATF6 acts as the intraluminal sensor while the amino terminus protrudes into the cytosol and functions as a basic leucine zipper (bZIP) transcription factor [37]. Dissociation of GRP78 from the luminal domain causes ATF6 to translocate to the Golgi apparatus. Located at the Golgi are site-1 and site-2 proteases, both of which have been implicated in the regulation of cholesterol metabolism [38]. It is here that ATF6 is cleaved, resulting in the release of the bZIP transcription factor into the cytosol [36]. From the cytosol, the processed ATF6 fragment localizes into the nucleus and helps upregulate the expression of genes responsible for mediating protein folding and ERAD [36, 39].

IRE-1α is a type 1 transmembrane protein containing an ER-sensing amino terminus, and a cytosolic carboxyl terminal endoribonuclease (RNase) and serine-threonine kinase domain [31, 40, 41]. Detection of unfolded proteins causes IRE-1α to dimerize and/or form higher order oligomers, which in turn activates its kinase domain. Subsequent trans and autophosphorylation stimulates the RNase activity of IRE-1α [42]. Acquisition of RNase catalytic activity enables for the excision of a 26-nucleotide intron within a mature X-box-binding protein 1 (XBP1) mRNA transcript in the cytosol [43]. The spliced XBP1 (sXBP1) mRNA encodes for an XBP1 isoform which, like ATF6, binds upstream cis-elements associated with chaperone and ERAD-mediated genes [39, 44]. Sustained ER stress augments the RNase activity of IRE-1α, thereby causing decreased specificity for XBP1 mRNA and elevated degradation of specific classes of mRNAs, 28S ribosomal RNA and microRNAs through regulated IRE1-dependent decay (RIDD) [45]. The degradation of RNA transcripts destined for the ER and ribosomal RNA is thought to initially help diminish mRNA translation to alleviate the protein load on the ER [46]. Nevertheless, prolonged RIDD activity resulting from chronic ER stress contributes to cell death by degrading ER-targeted mRNA that encode proteins involved in protein folding and anti-apoptotic microRNA, thus pushing cell fate toward apoptosis [47–49].

Lastly, PERK possesses type 1 transmembrane topology and a cytosolic kinase domain [31]. Structural analysis has revealed that the sensing luminal domain of PERK shares a conserved protein sequence with that of IRE-1α [26]. Unsurprisingly, both PERK and IRE-1α respond to ER stress in a similar manner. Along with IRE-1α, PERK indirectly reduces the quantity of unfolded polypeptide within the ER to allow for more efficient chaperone-mediated protein folding in a well-saturated ER lumen. The dimerization of PERK leads to the activation of its cytosolic kinases, which subsequently phosphorylate serine 51 on the α-subunit of eukaryotic initiation factor 2α (eIF2α) [26]. Phosphorylation of eIF2α prevents the translation of many nuclear-encoded mRNA transcripts by compromising the formation of the GTP·eIF2α·Met-tRNA_i_ ternary complex, which in turn prevents the assembly of the pre-initiation complex at the 5′ end of mRNA [50, 51]. Delaying translation re-initiation in this manner increases the probability that ribosomes will scan past inhibitory upstream open reading frames, resulting in increased translation of a specific subset of mRNAs, most notably mRNA that encodes ATF4 [50, 52]. Like ATF6, ATF4 is a bZIP transcription factor important for maintaining intracellular homeostasis through the upregulation of UPR-target genes involved in efficient protein folding, the antioxidant response and amino acid biosynthesis and transport [53]. In addition to promoting an adaptive response, ATF4 regulates the transcription of the gene encoding pro-apoptotic factor CCAAT-enhancer-binding protein homologous protein (CHOP) [54].

While the role of CHOP in stress-induced apoptosis remains obscure, it is thought that CHOP promotes apoptosis by 1) downregulating the expression of Bcl-2, a pro-survival proto-oncogene, 2) elevating the expression of pro-apoptotic BH3-only Bcl-2 family proteins such as Bad, Bim and p53 upregulated modulator of apoptosis and 3) coordinating intracellular calcium signaling [54, 55]. The latter relies on the involvement of the ER. In addition to its role in mediating stable protein folding, the ER serves an important function in cell signaling due to its ability to release calcium in response to second messengers. During unremitting PERK activation, CHOP accumulates to a point necessary to activate Ero1α, which drives the aperture of the ER calcium release channel inositol 1, 4, 5-triphosphate (IP3) receptor 1 [56, 57]. Prolonged efflux of calcium from the ER promotes the activation of calcium/calmodulin-dependent protein kinase II, which plays a role in promoting cell death [57]. Moreover, free cytosolic calcium leaks into the mitochondrial matrix, causing mitochondrial depolarization [58]. Mitochondrial uptake of calcium released from the ER also elevates the production of ROS through various mechanisms, including activating the mitochondrial permeability transition and stimulating Krebs cycle dehydrogenases [53, 58, 59]. Besides facilitating calcium release, Ero1α contributes to the production of hydrogen peroxide within the ER lumen [60].

Along with targeting Bcl-2 family genes and Ero1α, CHOP binds to promoter elements associated with growth arrest and DNA damage-inducible protein 34 (GADD34). The induction of GADD34 is imperative for attenuating signals downstream of the PERK-eIF2α-ATF4 pathway. This GADD34-dependent negative feedback loop relies on GADD34 recruiting protein phosphatase 1 (PP1) to dephosphorylate eIF2α. Mutating the conserved motifs important for binding PP1 in GADD34 impairs eIF2α dephosphorylation, thus supporting its regulatory role in mediating the phosphorylation state of eIF2α [61]. Moreover, knocking out CHOP diminishes GADD34 protein expression, leading to elevated levels of phosphorylated eIF2α when compared to wild-type (WT) cells experiencing ER stress [62]. Although GADD34-mediated dephosphorylation of eIF2α is essential for cells to restore global mRNA translation after acute insult, the overexpression of GADD34 increases the translation of mRNA transcripts induced during the later stages of prolonged ER stress, thereby elevating the protein load and restoring global translation of proteins involved in ROS production and apoptosis [63]. Additionally, GADD34 may have pro-apoptotic effects that are independent of its role in regulating eIF2α phosphorylation that contribute to ER stress-induced cell death [62, 64].

Another downstream effector of active PERK is the bZIP transcription factor nuclear factor-like 2 (Nrf-2), which is important for the expression of antioxidants [65]. Nrf-2 is normally sequestered within the cytosol by kelch-like ECH-associated protein 1 (Keap1) under basal states, however, the initiation of the UPR allows PERK to act on the Nrf-2-Keap1 complex. PERK-mediated phosphorylation of Nrf-2 promotes its dissociation from Keap1 and translocation into the nucleus where it upregulates the expression of genes essential for redox homeostasis. Cullinan et al. demonstrated that deleting Nrf-2 compromises the ability of mouse embryonic fibroblasts (MEFs) to cope with ER stress, as cells without Nrf-2 were more susceptible to undergoing ER stress-induced apoptosis compared to WT MEFs treated with tunicamycin, a pharmacological ER stress-inducing agent that blocks N-linked glycosylation [66]. The same study also provided evidence showing that PERK phosphorylation was sufficient to disrupt the Nrf-2-Keap1 complex, thereby allowing Nrf-2 to function as a transcription factor independent of the presence of ROS/RNS.

During the UPR, PERK and ATF6 signaling have been shown to upregulate the expression of sXBP1 mRNA (through different mechanisms) to produce an operative transcription factor responsible for inducing the expression of stress-response genes [67, 68]. Furthermore, there is evidence that the transcription of CHOP is also under the control of the active ATF6 transcription factor [69]. This demonstrates that not only do the ER sensors elicit independent signaling cascades in the face of ER stress, but there is cross-talk between the different UPR pathways in an effort to provide a robust response to physiological stress. In addition to ATF6 and IRE-1α regulating the transcription of chaperone proteins and enzymes mediating ERAD, both have also been implicated in the biosynthesis of ER phospholipids, which are used to expand the ER membrane, and in the regulation of other aspects of cellular metabolism [70, 71]. Interestingly, components of the UPR play an essential role in learning, memory and behavior. The eIF2α kinases, including PERK, regulate memory and synaptic plasticity by modulating gene expression and translation [72]. Moreover, a recent study demonstrated an important role for XBP1 in facilitating memory and long-term potentiation through the regulation of brain-derived neurotrophic factor expression [73]. The involvement of the UPR in optimizing the protein folding capacity of the ER as well as modulating cellular metabolism and cognitive function highlights the pleiotropic actions of the ER stress response in maintaining tissue and organismal homeostasis.

### UPR-mediated inflammatory pathways

**Fig. 3:**
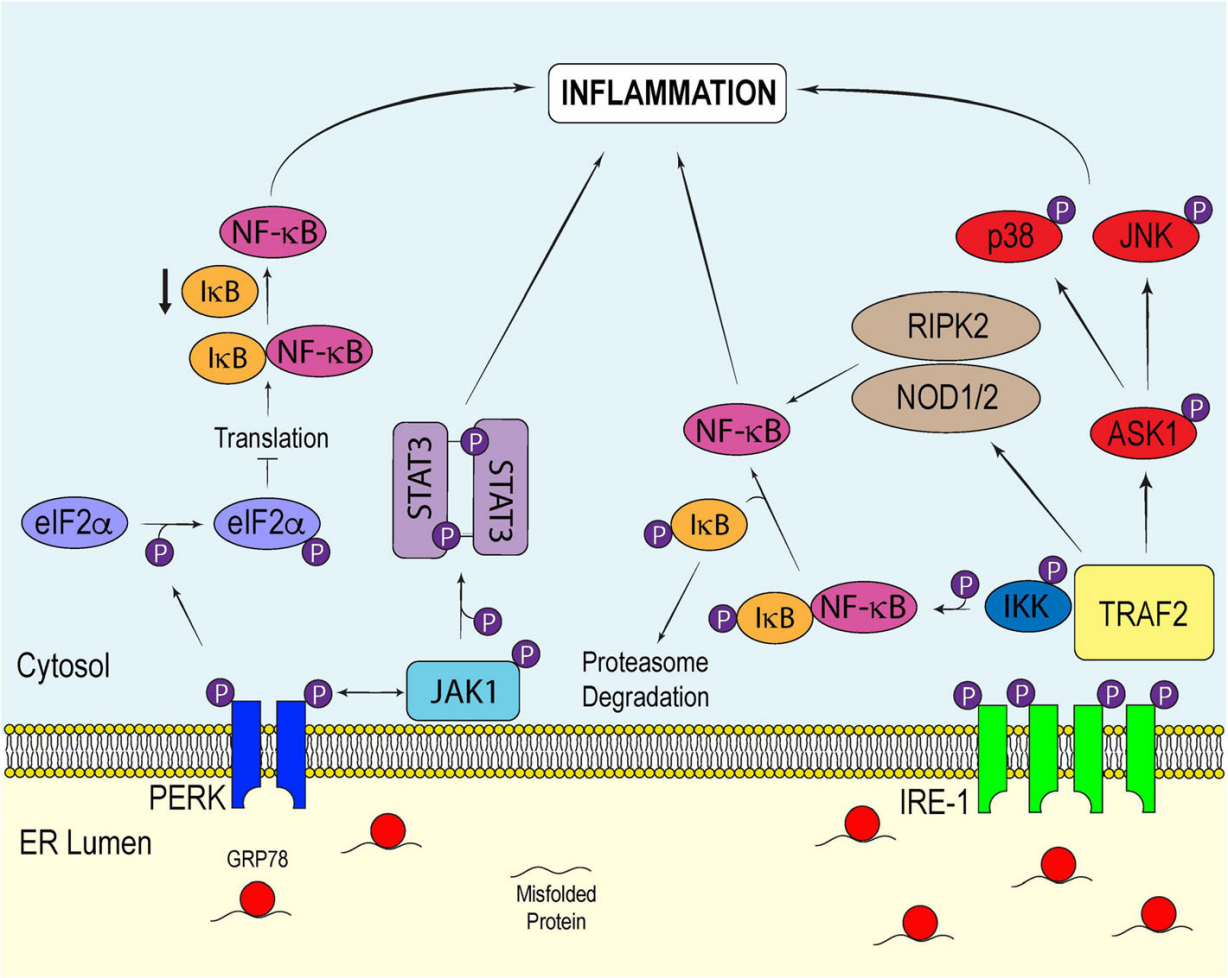
Inflammatory Pathways Induced by the UPR. The UPR stimulates various inflammatory pathways to alert surrounding cells of potential danger. The transient interaction between impaired proteostasis and inflammation is considered a beneficial feature of the UPR. Nevertheless, sustained UPR-induced inflammation is considered a pathological factor in many chronic disorders, such as neurodegenerative diseases. Inflammatory pathways associated with the UPR include the NF-κB, JAK1/STAT3, NOD1/2-RIPK2, JNK and p38 signaling pathways

In addition to coordinating the expression of stress-response genes during ER stress, the UPR initiates inflammatory pathways essential for the innate immune response (Fig. 3). The principal inflammatory signaling proteins activated during the UPR are the nuclear factor kappa-light-chain-enhancer of activated B cells (NF-κB) and the mitogen activated protein kinase (MAPK) family proteins c-Jun N-terminal kinase (JNK) and p38. It is important to note that NF-κB and the MAPKs regulate not only inflammatory gene expression, but they also play a role in mediating cell survival in a context-specific manner [74].

The NF-κB family of proteins are made up of homo- and heterodimeric transcription factors composed of proteins in the NF-κB/Rel family [75]. In unstressed cells, NF-κB is sequestered within the cytosol through physical interaction with inhibitors of κB (IκB). Signaling through the canonical NF-κB pathway activates the serine kinase IκB kinase (IKK), which is composed of two catalytic subunits (IKKα and IKKβ) and a regulatory subunit (IKKγ). Site-specific phosphorylation of IκB by IKK signals for its degradation through the ubiquitin-dependent recruitment of the 26S proteasome [76]. Subsequently, free NF-κB is able to localize to the nucleus and bind to κB sites in gene promoters, and drive the expression of cytokines and cell survival proteins. NF-κB can be activated by various forms of cell stress. For example, in addition to ER stress, elevated levels of cytosolic calcium and oxidative stress have been shown to promote NF-κB-mediated transcription [77, 78]. In the context of ER stress, the attenuation of global mRNA translation in response to eIF2α phosphorylation provides a means by which NF-κB is stimulated. Depressing mRNA translation decreases protein levels of IκB and NF-κB within the cytosol [79]. Because IκB has a shorter half-life than NF-κB, the higher proportion of NF-κB to IκB favors the migration of free NF-κB into the nucleus to upregulate the transcription of inflammatory genes.

Along with PERK, IRE-1α elicits inflammatory signaling during the ER stress response. After oligomerization, IRE-1α recruits the adaptor protein tumor-necrosis factor-α (TNF-α)-receptor-associated factor 2 (TRAF2), which couples the activation of IRE-1α with different inflammatory pathways. The formation of the IRE-1α-TRAF2 complex mediates cross-talk between active IRE-1α and the NF-κB and MAPK signaling pathways. TRAF2 directly interacts with IKK and indirectly with JNK by activating apoptosis signal-regulating kinase 1 (ASK1), which then coordinates the activation of JNK [20, 80, 81]. IRE-1α-mediated activation of IKK leads to the phosphorylation of IκB to promote NF-κB-dependent transcriptional regulation, while the IRE-1α-dependent activation of JNK stimulates the bZIP transcription factor activator protein 1 (AP-1). Thereafter, AP-1, a heterodimer composed of a differential combination of Fos, Jun, ATF and Maf sub-family members, binds to enhancer elements which upregulate the transcription of inflammatory genes [82].

Interestingly, the IRE-1α-TRAF2 axis has been shown to stimulate the nucleotide-binding oligomerization domain 1 and 2 (NOD1/2)-receptor-interacting serine/threonine-protein kinase 2 (RIPK2) pathway, resulting in the activation of NF-κB [83]. This proposed mechanism was supported in an in vivo murine model of *Brucella abortus* infection. *Brucella abortus* induces ER stress by injecting host cells with the VceC virulence factor via its type IV secretion system. Here, Keestra-Gounder et al. demonstrated that the resulting ER stress-induced production of interleukin (IL)-6 in infected mice was dependent on TRAF2, NOD1/2 and RIPK2 interplay. These findings provided further evidence of dynamic interactions between innate immunity and UPR-induced inflammation.

In conjunction with its involvement in initiating inflammation, IRE-1α can facilitate cell death through its interactions with the apoptotic proteins during ER stress [84]. IRE-1α-dependent activation of caspase-12 has been reported to be a dispensable contributor in the execution of ER stress-induced apoptosis in mice and rats [85–87]. Nevertheless, many human variants of caspase-12 possess loss-of-function mutations that promote the synthesis of a truncated protein without functional activity, and thus may not be a significant contributor to ER stress-induced cell death in humans [87]. The IRE-1α-TRAF2-JNK pathway coordinates cell death by facilitating Bax-dependent apoptosis and inhibiting the pro-apoptotic protein Bcl-2, while the IRE-1α-TRAF2-p38 branch may enhance CHOP transcriptional activity [20, 88, 89]. This understanding highlights the importance of TRAF2 in linking the UPR to a diverse range of signaling pathways to trigger the appropriate physiological response.

Recently, the interaction between PERK and Janus kinase 1 (JAK1) in the UPR was elucidated in astrocytes. It has been recognized that ER stress influences the JAK-signal transducers and activators of transcription (STAT) pathway [90, 91], however, the molecular mechanisms underlying its involvement in the context of neurodegeneration and how it alters the JAK-STAT pathway in glial cells remained to be clarified. We observed that JAK1-STAT3 signaling in ER stressed astrocytes was dependent on PERK [92]. Transfecting astrocytes with PERK small interfering RNA, followed by treatment with thapsigargin, a non-competitive inhibitor of the sarco/endoplasmic reticulum Ca^2+^ ATPase used to induce ER stress, attenuated JAK1 and STAT3 phosphorylation. Additionally, PERK knockout MEFs incubated with thapsigargin expressed significantly less phosphorylated STAT3 and STAT3-dependent inflammatory cytokines and chemokines relative to their WT counterparts. Mass spectrometry revealed that JAK1 phosphorylates PERK at tyrosine 585 and 619 in vitro. While further investigation is needed to completely unravel how STAT3 is phosphorylated by the PERK/JAK1 complex, these findings present a novel pathway implicating the UPR in driving neuroinflammation.

Each of the three ER stress sensors serves a multifunctional role in maintaining ER protein homeostasis under transient ER stress. If the cell is unable to ameliorate intrinsic protein misfolding stress, the cell will induce apoptotic pathways to avoid continuously secreting distress signals to neighboring cells. The category of stimuli or environmental conditions may be an important determinant regarding whether the cell will trigger a coordinated cell death. One must also consider that certain cell types, such as highly secretory cells, must constantly maintain an optimal ER protein folding environment, making them more susceptible to ER stress.

Chronic ER stress leads to the disproportionate activation of the ATF6, IRE-1α and PERK pathways to amplify the apoptotic component of the UPR [93]. Some experimental models respond to severe ER stress by attenuating ATF6 and IRE-1α signaling and augmenting PERK activation to allow apoptotic signals to dominate [93]. Because CHOP possesses a short half-life, chronic PERK activation is required to overwhelm the adaptive signals of the UPR to promote cell death [94, 95]. Similarly, sustained IRE-1α signaling has the potential to initiate apoptosis in other situations. While various pharmacological approaches have provided invaluable insights into the physiologic role of the UPR, more work must be done to fully appreciate how each of the branches of the UPR respond to specific stimuli and how they integrate to mediate apoptotic events.

## ER stress in neurodegenerative diseases

The activation of the UPR plays an essential role in maintaining vital biological processes within the brain during cellular stress. In fact, moderate ER stress enhances cellular protection against subsequent insult by altering the transcriptome and proteome of the cell to increase the adaptive capacity of the ER, a response called the hormetic response [9, 96–99]. However, prolonged ER stress developed in neurodegenerative diseases is believed to disrupt the protective mechanisms of the UPR, leading to the activation of inflammatory and apoptotic programs that promote neurotoxicity. In the following sections we will briefly describe the mechanisms underlying how ER stress is generated in neurodegenerative diseases, such as Alzheimer’s disease (AD), Parkinson’s disease (PD), Amyotrophic Lateral Sclerosis (ALS) and Multiple Sclerosis (MS), then address its potential contribution to the development of pathological neuroinflammation. In general, ER stress is a consequence of disturbances in protein-quality control machinery, ER Ca^2+^ dysregulation, protein-trafficking impairment or direct defects in UPR components [9].

### Alzheimer’s disease

AD is a common age-dependent neurodegenerative disease that accounts for a significant number of reported dementia cases [100]. The pathology of AD is characterized by the formation of intracellular neurofibrillary tangles (NFTs) composed of hyperphosphorylated tau and the extracellular parenchymal deposition of amyloid-β (Aβ) aggregates [101, 102]. The cytoplasmic protein tau normally serves to stabilize microtubules which form ‘tracks’ that facilitate intracellular vesicle trafficking and axonal elongation and maturation. This is highlighted by the finding that knocking down tau leads to severe neurite growth defects in primary cerebellar neurons [103]. However, certain insults cause an imbalance between the activities of tau kinases and phosphatases that lead to the abnormal phosphorylation of tau [104]. In its hyperphosphorylated state, tau becomes soluble and, in turn, polymerizes to form oligomers and/or NFTs [105]. In the case for Aβ pathology, genetic studies have implicated mutations in Aβ precursor protein (APP) and in the transmembrane proteins presenilin-1 (PS1) and presenilin-2 (PS2), which act as subunits for the γ-secretase complex, as the predominant genetic factors contributing to the onset of familial AD [106, 107]. Potentially pathological Aβ is liberated when APP is sequentially cleaved at the plasma membrane by β-secretase, then γ-secretase. [101]. This leads to an extracellular accumulation of either total Aβ or increase relative concentrations of amyloidogenic Aβ, such as Aβ42. Impaired clearance of Aβ has also been implicated in AD, as it creates an imbalance of its turnover in the brain [108].

Chronic ER dysfunction is highly associated with the memory and cognitive manifestations commonly observed in different experimental models of AD [109, 110]. To this point, Ma et al. elucidated that selectively abating the expression of PERK in mice possessing AD-linked mutations in genes encoding APP and PS1 prevented the aberrant phosphorylation of eIF2α [111]. This, in turn, improved synaptic plasticity and spatial memory in AD mice, consistent with the requirement for active protein translation in memory consolidation [112]. Interestingly, sXBP1 overexpression ameliorates cognitive function in the 3× Tg AD mouse model [113]. The eIF2α kinases general control non-derepressible 2 (GCN2) [111] and double stranded RNA-dependent kinase (PKR) have also been implicated in memory impairment [110, 114]. Multiple studies have demonstrated that Aβ oligomers can activate PKR and induce ER stress by eliciting the TNF-α pathway [110, 115]. Additionally, Aβ may stimulate ER Ca^2+^ release through ryanodine receptors and IP3 receptors, which triggers ER stress, neuronal apoptosis and mitochondrial fragmentation [116–118]. Inhibition of both GCN2 and PKR through different mechanisms significantly improves cognitive function in murine AD models [111, 114]. These findings suggest that pathophysiological conditions, not just ER stress, which lead to sustained eIF2α phosphorylation have the potential to aggravate the cognitive abnormalities seen in AD.

Abnormal protein aggregates interfere with the normal processes involved in protein maintenance and trafficking in models of neurodegeneration. Regarding AD, soluble tau has been shown to cause pathological ER stress by targeting and impairing components involved in ERAD [119]. Paradoxically, pre-existing ER stress also promotes NFT formation. It is well known that Aβ oligomer-dependent ER stress responses can lead to the activation of different kinases, such as the serine/threonine kinase glycogen synthase kinase 3 (GSK-3) [120, 121]. This kinase (among others) is capable of subsequently phosphorylating specific epitopes on tau that contribute to the development of NFT [120, 121]. Therefore, ER stress and hyperphosphorylated tau could be induced by each other in a cycle to propagate AD pathology [122]. More recently, however, the correlation between NFT formation and AD severity had been scrutinized [123]. It seems now that soluble oligomers of tau and Aβ may be the primary neurotoxic agents that contribute to AD [123].

It has been suggested that familial AD-linked PS1 mutations suppress the activation of IRE-1α. This predisposes cells to become more susceptible to ER stress due, in part, to decreases in protein chaperone synthesis as a result of reduced UPR induction [124]. A study using SK-N-SH cells and fibroblasts expressing a PS1 mutant associated with familial AD demonstrated that mutant PS1 also disrupts PERK activation, potentially in a similar manner as IRE-1α, and delays nuclear accumulation of processed ATF6 in response to ER stress [125]. The aberrantly spliced isoform of PS2 (PS2V) is also linked to AD. Similar to the PS1 mutations, this isoform increases the vulnerability of the cell to ER stress [126]. Alternatively, the over-expression of PS1 and PS2 mutants in cells perturbs ER calcium homeostasis, implying another mechanism by which genetic mutations in the presenilin genes contribute to AD [127–129]. With this in mind, there is contradictory evidence indicating that neither ablation of PS1 or expression of familial AD-linked PS1 variants impairs the expression of GRP78 mRNA and the activation of IRE1-α [130]. Because of limited knowledge regarding how ER stress is generated during AD, more investigation is needed to fully appreciate how dysregulated UPR signaling contributes to the pathology of AD.

### Parkinson’s disease

PD is a chronic and progressive movement disorder characterized by the selective loss of dopaminergic neurons in the substantia nigra, and the presence of intraneuronal filamentous inclusion bodies called Lewy bodies. While the development of Lewy bodies is not a definitive causative factor, they are deemed to be a pathological hallmark of PD. A post-mortem study showed that the percentage of Lewy body-containing dopaminergic neurons positive for caspase-3 is significantly higher than the percentage of caspase-3–positive dopaminergic neurons without Lewy bodies, indicating that Lewy body-containing dopaminergic neurons are predisposed to undergo apoptosis [131]. A principal component of Lewy bodies in PD are the abnormal filaments of α-synuclein, which seem to form due to different genetic factors, such as the multiplication of the SCNA locus, or non-genetic factors, such as aberrant post-translational modifications [132–134].

Common mutations implicated in autosomal recessive PD reside within the *Parkin* gene, which encodes for an E3 ubiquitin ligase that is necessary for mitophagy [135, 136]. Studies using post-mortem brain samples and mouse models also suggest that Parkin can be inactivated by post-translational modifications, such as oxidation, nitrosylation and the addition of dopamine [135]. Disrupting the E3 ligase activity of Parkin or defects in PTEN-induced kinase 1, which recruits Parkin to the outer membrane of damaged mitochondria, is thought to play a critical role in the development of familial and sporadic PD, mainly through its failure to maintain mitochondrial fidelity [137]. Similarly, mutations within the gene that encodes leucine-rich repeat kinase 2 (LRRK2) have been shown to promote PD [138]. LRRK2 is a widely expressed protein important for regulating various biological processes. Mutant LRRK2 is highly associated with the onset of inherited and sporadic PD, and the resulting LRRK2-mediated toxicity may be dependent on its kinase activity [139]. Nevertheless, the mechanisms underlying its role in the pathogenesis of PD are still being unraveled.

The notion that prolonged ER stress contributes to PD pathology was first supported with the findings that neurons in toxin-induced models of PD highly expressed genes involved in the UPR [140]. It is now appreciated that α-synuclein-induced neurotoxicity may result from nitrosative stress, accumulation of ERAD substrates and/or defective vesicular trafficking, all of which can lead to ER stress [141]. To this point, under conditions of nitrosative stress, S-nitrosylation directly inactivates PDI [142]. This inactivation impairs proper protein folding and hinders PDI-mediated attenuation of neuronal cell death [142]. Moreover, the concomitant accumulation of toxic α-synuclein oligomers in the ER further exacerbates the severity of ER stress, leading to deleterious UPR signaling [134, 143]. Some findings indicate that α-synuclein-dependent ER stress is the result of blocking ER to Golgi vesicular trafficking, as preventing vesicle mobilization from the ER causes the accumulation of protein cargo within the ER lumen [144]. Targeting pathways associated with these abnormal phenotypes through pharmacological intervention in vitro has been shown to rescue neuronal loss observed in PD models [141].

Post-mortem analysis revealed that human PD patients exhibited greater phosphorylated PERK and eIF2α in neuromelanin containing dopaminergic neurons relative to control cases [145]. In the same study, phosphorylated PERK colocalized with α-synuclein within dopaminergic neurons derived from PD patients. PC12 cells possessing the A53T mutation in the α-synuclein gene, a point mutation that increases the tendency of α-synuclein to form amyloid-like fibrils, exhibit elevated levels of phosphorylated eIF2α, CHOP, GRP78 and active caspase-12 [146]. Treatment with the caspase inhibitor z-VAD or salubrinal, which prevents the de-phosphorylation of eIF2α, improved cell viability of A53T PC12 cells by attenuating apoptotic signaling [146]. Taken together, these findings suggest that pathological α-synuclein may exacerbate disease progression by promoting excessive or unmitigated ER stress responses.

Stress-induced Parkin expression serves as a protective mechanism elicited by the UPR [147, 148]. The use of chromatin immunoprecipitation led to the discovery that ATF4 regulates Parkin gene expression by binding to CREB/ATF sites in the *Parkin* promoter [148]. The resulting increase in Parkin protein protects against ER stress-induced cell death in neurons by preventing the toxic accumulation of Parkin substrates. Moreover, the protective function of Parkin could be partially explained with the discovery that Parkin promotes the production of sXBP-1, which upregulates the transcription of pro-survival genes [149]. Recent evidence indicates that Parkin controls the function of PS1 and PS2, suggesting a possible link between defective Parkin and the pathogenesis of both AD and PD [150]. LRRK2 also helps maintain neuronal integrity against induced Parkinsonism by alleviating the consequences of ER stress. Yuan et al. demonstrated that LRRK2 saves neuroblastoma cells and *C. elegans* dopaminergic neurons from 6-OHDA or α-synuclein toxicity [151]. They also demonstrated that loss of function mutations in LRRK2 compromises the expression of GRP78, resulting in the hyperactivation of p38 and elevated neuronal death. Collectively, impairment in these protective mechanisms in neurons provides an alternative disturbance that contributes to the progression of PD.

### Amyotrophic lateral sclerosis

ALS is a progressive neurodegenerative disease characterized by the destruction of motor neurons, which leads to paralysis and poor patient prognosis [152]. Among cases of ALS, 10% are considered familial, while the remaining 90% of cases are sporadic [153]. A pathological hallmark of familial ALS is the formation of ubiquitinated cytoplasmic inclusions composed of misfolded superoxide dismutase-1 (SOD1) [154]. However, defects in the *SOD1* gene only account for 20% of familial ALS cases, and 2% of sporadic cases [155, 156]. An accrual of evidence now connects mutations in genes encoding chromosome 9 open reading frame 72 (C9orf72), transactive response DNA binding protein 43 (TDP43), and Fused in Sarcoma RNA-binding protein (FUS) (among others) to ALS pathology [155, 157–159]. In all, a large proportion of genetic alterations implicated in ALS promote disease onset and progression by either perturbing protein quality control mechanisms, RNA integrity or cytoskeletal dynamics [155]. As in other mutations associated with neurodegenerative diseases, ALS-associated mutations are expressed ubiquitously within the CNS (neurons and surrounding neuroglia), with strong evidence that both cell-autonomous and -nonautonomous mechanisms contribute to the progressive loss of motor neurons [155].

Mediators associated with the UPR are upregulated in the spinal cords of ALS patients and in mutant SOD1 transgenic mice [160–162]. For instance, CHOP is highly expressed in motor neurons, glial cells and spinal cords of mutant SOD1 transgenic mice [163]. A similar observation is seen in spinal cord samples of sporadic ALS patients [163]. ERAD impairment is considered a central mechanism by which mutant SOD1 induces ER stress in ALS. Here, mutant SOD1 protein has been shown to inhibit a specific component of the retro-translocation machinery involved in ERAD called Derlin-1 by directly interacting with its cytoplasmic C-terminus [164]. Failure to export misfolded substrates from the ER in NSC34 cells leads to their accumulation within the ER lumen, which promotes neuronal death by eliciting the IRE-1-TRAF2-ASK1 pathway [164].

Increased motor neuron loss and SOD1 aggregation is observed in SOD1^G85R^ PERK^+/−^ mice compared to SOD1^G85R^ mice fully expressing PERK [165]. Interestingly, ATF4 deficiency in SOD1^G85R^ mice exacerbates SOD1 aggregation, but delays disease onset and reduces the expression of pro-apoptotic genes [166]. XBP1-null NSC34 motor neurons expressing mutant SOD1 are more apt to clear mutant SOD1 aggregates [167]. Moreover, silencing XBP1 in vivo provides protection against disease progression in mutant SOD1 mice [167]. Taken together, there is contradictory evidence regarding the protective effects of the UPR in experimental models of ALS, suggesting that the extent to which the UPR contributes to ALS is context-dependent.

PDI has been shown to be upregulated in SOD1^G93A^ ALS rats and mice [168]. Furthermore, post-mortem human brain samples exhibit greater PDI expression in comparison to controls, implying that PDI is induced in response to the abnormalities associated with ALS [169]. The protective role of PDI in ALS emanates from its ability to facilitate folding of misfolded assemblies, thereby reducing SOD1 aggregate-mediated toxicity [169]. As seen in PD, PDI expressed in spinal cords of ALS patients is highly S-nitrosylated [170]. Increased RNS production has been reported in ALS, and the resulting nitrosative stress may impair the function of PDI through this post-translational modification [171].

Aggregates composed of mutant TDP-43, FUS or C9orf72 also initiate the UPR program [172–174]. To this point, overexpressing ALS-associated mutant TDP-43 in Neuro2a neuroblastoma cells results in greater induction of CHOP, XBP1 and ATF6 [173]. Moreover, mutations in FUS contribute to the formation of cytoplasmic protein inclusions that trigger ER stress responses in NSC34 motor neurons, and are found to co-localize with PDI in post-mortem spinal cord samples from ALS patients [175, 176]. Lastly, a study expressing poly(GA) repeats in neuronal cultures, which model ALS-associated repeat expansions in the C9orf72 gene, contribute to neuronal death by inducing ER stress [172]. When treated with salubrinal or the chemical chaperone TUDCA, these neurons are rescued from ER stress-mediated cell death, indicating that mutations in the C9orf72 gene contribute to neurotoxicity by promoting ER dysfunction [172]. Overall, these findings highlight how pathological assemblies implicated in ALS contribute to motor neuron loss. Even with the present understanding that SOD1-linked mutations only account for a relatively small proportion of ALS cases, many studies investigating the relationship between ER stress and ALS largely utilize animal models expressing mutant SOD1. Therefore, it will be of importance to further elucidate the mechanisms by which ER stress is generated in other ALS models in order to fully grasp how ER stress aggravates ALS pathology.

### Multiple sclerosis

MS is T lymphocyte-mediated autoimmune disease characterized by the spatiotemporal dissemination of white matter lesions within the CNS [177]. While the etiology of MS remains in question, it is thought to be initiated by autoreactive T lymphocytes that have breached the blood brain barrier (BBB) or the blood-cerebral spinal fluid-barrier and have mounted an autoimmune response directed toward self-CNS antigens [178]. Autoreactive B cells and innate immune cells, such as NK cells, have also been reported to localize to the CNS from the periphery during MS pathology [179]. In the early stages of MS, peripheral humoral and innate immune cells accumulate in the perivascular and ventricular spaces that separate the blood vessels from the adjacent brain tissue, reactivated by local antigen presenting cells and subsequently move into the brain parenchyma to promote severe neuroinflammation [180]. These reactive immune cells release a plethora of inflammatory mediators, including nitric oxide, ROS and inflammatory cytokines, which impair neuronal function and activates CNS-resident astrocytes and microglia. Together, the continuous secretion of soluble inflammatory mediators promotes the development of a neurotoxic microenvironment that facilitates demyelination, axonal degeneration and oligodendrocyte and neuronal death.

One explanation for the development of autoreactive T and B cells is that some viral antigens presented by major histocompatibility complex II in the periphery or the CNS share homology with that of myelin components. Effector lymphocytes that enter the perivascular space are reactivated by antigen presenting cells presenting myelin peptides that share sequence and structural similarities with foreign-peptides [181]. This phenomenon, molecular mimicry, is considered a potential mechanism by which pathogens break self-immunological tolerance and induce an autoimmune reaction. Pathogens sharing high degrees of peptide similarity with myelin-derived peptides include Human Herpes virus type 6 and Epstein Barr virus [182]. The inflammatory milieu brought about by infiltrating innate immune cells and reactive T lymphocytes in the initial stages of the disease promotes further T cell polarization to the T_H_1 or T_H_17 subsets to amplify neuronal damage. From a genetic standpoint, single polymorphisms within specific candidate genes increase the susceptibility of individuals to developing MS. Such candidate genes may include genes located within the human leukocyte antigen (HLA) locus and immunological non-HLA genes involved in central tolerance, cytokine production and homeostatic proliferation [177].

Real time qPCR analysis of CNS tissue from MS patients has revealed that the ER stress markers ATF4, GRP78 and CHOP are significantly upregulated in the white matter of MS patients relative to tissue from non-MS individuals [183]. In agreement with these findings, a study performing detailed semiquantitative immunohistochemical and molecular analysis on multiple CNS cell-types in active MS lesions found that GRP78 and CHOP were highly upregulated in astrocytes, microglia and oligodendrocytes [184]. The elevated expression of UPR markers in MS lesions points toward a possible link between impaired ER proteostasis and the development of active lesions.

There are multiple potential events hypothesized to induce ER stress during MS. Glutamate excitotoxicity is an important mechanism that contributes to autoimmune demyelination and lesion formation [185]. Glutamate induces the expression of GRP78, and GRP78 knockdown leads to a significant increase in excitotoxicity-induced apoptosis [186]. This suggests that glutamate excitotoxicity promotes neuronal death through an ER stress-dependent mechanism, and the upregulation of GRP78 helps neurons cope with the excessive amounts of glutamate. In accordance, GRP78 seems to be vital for maintaining cell survival during MS. Oligodendrocyte-selective heterozygous deletion of GRP78 in mice induced with experimental autoimmune encephalomyelitis (EAE), an experimental model used to mimic the symptoms of MS, aggravates disease severity and enhances oligodendrocyte death [187].

Hypoxia is another potential ER stress inducer that is characteristic in, though not restricted to, MS. Histological evidence points toward a similar hypoxic-type response in diseased tissue of MS patients, as the hypoxia-related antigen D-110 is strongly expressed in tissue also expressing high levels of CHOP [184]. Alternatively, expression of human endogenous retrovirus (HERV) envelop proteins may contribute to the pathology of MS by initiating neuroinflammatory and ER stress responses in the brain [12, 188]. For instance, the overexpression of the HERV envelope glycoprotein Syncytin-1 causes astrocytes to upregulate ER stress responses and the production of proinflammatory mediators that promote oligodendrocyte toxicity [12]. Finally, the inflammatory environment in the CNS could trigger ER stress in highly myelinating cells, such as oligodendrocytes. Due to their high demand for lipid synthesis, mature oligodendrocytes are more susceptible to ER stress when exposed to high levels of proinflammatory mediators. It was previously demonstrated that interferon (IFN)-γ drives ER stress and cell death in oligodendrocytes both in vitro and in vivo [189]. In this same study, mice that were haploinsufficient for PERK were more susceptible to forced expression of IFN-γ, leading to myelination defects and oligodendrocyte death. Therefore, excessive neuroinflammation may induce ER stress in myelinating cells which would not only disrupt their ability to myelinate neuronal axons, but can also lead to cell death.

### ER stress-linked inflammation in neurodegenerative diseases

The development of ER stress is considered an underlying factor contributing to the clinical manifestations linked to many neurodegenerative disorders. In addition to the diseases previously described, pathological processes associated with other neuropathologies, such as prion diseases [190–193], human immunodeficiency virus associated-neurocognitive disorders (HAND) [193, 194] and a variety of lysosomal storage diseases [195], promote cellular and physiological challenges which perturb ER homeostasis. A unifying feature of all of these diseases is the presence of neuroinflammation [2, 196–198]. While few studies have directly examined the interactions between ER stress and inflammation in the CNS, there is evidence that these processes are intimately linked [24, 199, 200].

In brain tissue, microglia and astrocytes collaborate to mediate inflammation by integrating environmental information and carrying out an appropriate response. Microglia are CNS-resident phagocytic cells derived from the yolk sac. These sentinels of the CNS are the principal innate immune cell in the brain and have a key role in orchestrating inflammatory responses [201–203]. Astrocytes are also considered important regulators of the CNS, as they assist in neuronal metabolism, synaptic transmission, lay down the barriers isolating the neural tissue of the brain and coordinate the finely-tuned events of neuroinflammation along with microglia [3]. These glial cells possess a diverse repertoire of innate receptors, such as scavenger receptors and pattern recognition receptors, which allow them to augment the expression of inflammatory cytokines and chemokines under metabolic stress or interaction with PAMPs or DAMPs [204, 205].

Extracellular protein aggregates or oligomers underlie the pathology of various neurodegenerative disorders, and act as “danger signals” released from apoptotic or necrotic neurons [2]. These pathological assemblies can be recognized by innate immune receptors residing on neighboring glial cells [2, 206]. For example, Aβ oligomers are perceived to act as ligands for both the TNF-α receptor and toll-like receptor 4 [207]. Transient substrate-receptor interaction promotes an inflammatory response that initiates debris clearance via phagocytosis by microglia [2]. However, chronic exposure to these DAMPs or internalization of abnormal protein aggregates alters the functional properties of immunocompetent microglia and astrocytes to promote a reactive phenotype [2, 208]. In MS, autoreactive peripheral immune cells initiate an inflammatory response against myelin-derived antigen and promote neurotoxicity not only by compromising neuronal integrity directly, but causing astrocytes and microglia to secrete cytokines and other inflammatory mediators that contribute to demyelination [2, 209].

While chronic ER stress in neurons largely triggers signals to initiate apoptosis, extensive ER stress in glial cells has the potential to promote an inflammatory microenvironment characteristic in neurodegenerative diseases. Consistent with the role of astrocytes in mediating immunological homeostasis through its interactions with other cell types, the ER stress-induced upregulation in astrocytic inflammatory processes can encourage an inflammatory M1-like phenotype in microglia [92]. Similarly, neuronal ER stress has been shown to be positively correlated with microglial activation in a traumatic brain injury rat model [210]. ER stress not only influences pathways that result in the production of inflammatory mediators, but it also alters the responsiveness of cells to immunogenic stimuli. To this point, it has been documented that the administration of both prostaglandin E_2_ and IFN-γ synergizes with ER stress to increase the production of IL-6 in glial cells [211]. Likewise, TNF-α autocrine signaling during ER stress significantly enhances the apoptotic signals of the UPR [80].

PERK knockdown experiments suggest that the association between ER stressed astrocytes and microglia activation is initially dependent on PERK signaling in astrocytes [92]. PERK haploinsufficiency and partial PERK inhibition using the small molecule PERK inhibitor GSK2606414 selectively attenuates the production of ER stress-induced inflammatory cytokines and chemokines, including IL-6, C-C Motif Chemokine Ligand (CCL)2 and CCL20 [212]. Interestingly, treating ER stressed astrocytes with ISRIB, a compound which reverses the translational block of phosphorylated eIF2α, attenuates ER stress-induced inflammatory gene expression [212]. We propose that the inflammatory signals induced during ER stress in astrocytes significantly relies on PERK-dependent eIF2α phosphorylation. These beneficial outcomes of PERK-eIF2α modulation fall in line with previous studies demonstrating that treating prion-diseased mice with GSK2606414 or ISRIB confers neuroprotection by partially recovering global translation rates [213, 214]. Conversely, preventing eIF2α de-phosphorylation in response to tramatic brain injury using salubrinal is beneficial and attenuates neuroinflammation [11]. While it is becoming clear that PERK signaling has an important role in the regulation of neuroinflammation and neurodegeneration, a more complete understanding of the PERK-eIF2α pathway is needed to define the context and cell-specific roles. Therefore, manipulating the PERK-eIF2α axis without disturbing its homeostatic function could present an unappreciated way to alleviate aberrant neuroinflammation.

## Conclusion

Many fundamental questions remain regarding the role of inflammation and ER stress in neurological diseases. Is inflammation beneficial or detrimental in neurodegenerative diseases? Most likely that it is important for tissue repair and neural regeneration, but detrimental when dysregulated. To complicate matters, the UPR system may be helpful or harmful depending on the level and spatial-temporal occurrence of ER stress. Cross-talk between the two programs may have beneficial functions through reciprocal regulation that promotes protective immunity. However, ER stress-induced amplification of inflammation may worsen chronic diseases [215].

Our understanding on if and how ER stress directly provokes an inflammatory reaction in neurodegenerative diseases remains to be clarified. Studies from our laboratory demonstrate that ER stress generated in murine astrocytes encourages PERK-dependent inflammatory signaling in vitro, suggesting that astrocytes themselves are potential contributors to neurotoxic inflammation in the face of ER dysfunction [92, 212]. Nevertheless, the relevance of these findings as it pertains to animal models and patients remains to be determined. Further, whether microglia respond to ER stress in the same vain has yet to be explored. Investigators must be cognizant of how agents used to manipulate the UPR will impinge on its homeostatic roles when devising pharmacological approaches to treat neurodegenerative diseases. Moreover, since both defective and chronic UPR signaling contribute to neuronal death in disease, developing agents which strictly attenuate pathways elicited by the ER stress response are insufficient. It is likely that targeting specific signaling components of the UPR that are predicted to enhance the pro-survival signals of the UPR or attenuate its inflammatory/apoptotic responses will possess more favorable outcomes.

In summary, ER stress and neuroinflammation are common pathological features of neurodegenerative diseases, and the mechanisms by which they interact during neurodegeneration remain to be elucidated. Further knowledge of this cross-talk will help us understand whether targeting cell stress pathways, such as ER stress in neurodegeneration, can control aberrant neuroinflammation and treat neurological disorders. To date, many studies have demonstrated beneficial effects of modulating ER stress pathways either genetically or pharmacologically in model organisms. However, the looming question remains: will targeting the UPR pathways be safe and beneficial in patients?

## Abbreviations

AD: Alzheimer’s disease
ALS: Amyotrophic Lateral Sclerosis
AP-1: Activator protein 1
APP: Aβ precursor protien
ASK1: Apoptosis signal-regulating kinase 1
ATF: Activating transcription factor
Aβ: Amyloid-β
BBB: Blood brain barrier
BiP: binding immunoglobulin protein
bZIP: basic leucine zipper
CHOP: CCAAT-enhancer-binding protein homologous protein
CNS: Central nervous system
DAMP: danger-associated molecular patterns
eIF2α: eukaryotic translation initiation factor 2α
ER: Endoplasmic reticulum
ERAD: ER-associated degradation
Ero1α: ER oxidoreductase α
FAD: Familial Alzheimer’s disease
GADD34: Growth arrest and DNA damage-inducible protein 34
GCN2: General control non-derepressible 2
GRP: Glucose-regulated protein
GSK-3: Glycogen synthase kinase 3
HERV: human endogenous retrovirus
HLA: Human leukocyte antigen
IFN-γ: Interferon-γ
IKK: IκB kinase
IL: Interleukin
IRE-1α: Inositol-requiring 1α
IκB: inhibitors of κB;
JAK1: Januse kinase 1
JNK: c-Jun N-terminal kinase
Keap1: Kelch-like ECH-associated protein 1
LRRK2: Leucine-rich repeat kinase 2
MAPK: Mitogen activated protein kinase
MEF: Mouse embryonic fibroblasts
MS: Multiple Sclerosis
NFT: Neurofibrillary tangle
NF-κB: Nuclear factor kappa-light-chain-enhancer of activated B cells
NOD1/2: Nucleotide-binding oligomerization domain 1/2
Nrf-2: Nuclear factor-like 2
PAMP: pathogen-associated molecular patterns
PD: Parkinson’s disease
PDI: Protein disulphide isomerase
PERK: Double-stranded RNA dependent protein kinase-like ER kinase
PKR: Double stranded RNA-dependent kinase
PPI: Protein phosphatase 1
PS: Presenilin
RIDD: Regulated IRE1-dependent decay
RIPK2: Receptor-interacting serine/threonine-protein kinase 2
RNase: Endoribonuclease
RNS: Reactive nitrogen species
ROS: Reactive oxygen species
SRP: Signal-recognition particle
STAT: Signal transducers and activators of transcription
sXBP1: Spliced x-box-binding protein 1
TNF-α: Tumor-necrosis factor-α
TRAF2: TNF-α-receptor-associated factor 2
UPR: Unfolded protein response
WT: Wild-type
XBP1: X-box-binding protein 1
